# The effect of a health literacy approach to counselling on the lifestyle of women with gestational diabetes: A clinical trial

**DOI:** 10.12688/f1000research.13838.1

**Published:** 2018-03-06

**Authors:** Mehrafza Gharachourlo, Zohreh Mahmoodi, Mahnaz Akbari Kamrani, Maryam Tehranizadeh, Kourosh Kabir

**Affiliations:** 1 Student Research Committee, Alborz University of Medical Sciences, Karaj, Iran; 2 Non-Communicable Diseases Research Center, Alborz University of Medical Sciences, Karaj, Iran; 3 Social Determinant of Health Research Center, Alborz University of Medical Sciences, Karaj, Iran; 4 Psychology Department, Payame Noor University, Kara, Iran; 5 Clinical Research Center Unit, Madani Hospital, Alborz University of Medical Sciences, Karaj, Iran

**Keywords:** Gestational diabetes, health literacy, lifestyle, counselling

## Abstract

**Background:** Gestational diabetes is a common pregnancy disorder that affects the mother’s and neonate’s health. The present study was conducted to investigate the effect of a health literacy approach to counselling on the lifestyle of women with gestational diabetes. The present randomized controlled clinical trial was conducted in 2017 using a parallel design. The subjects included 84 eligible women presenting to Alborz and Kamali Hospitals, Karaj, Iran.

**Methods:** Convenience sampling was first used to select the subjects. They were then assigned to an intervention or control group based on randomized blocks of four. Both groups attended counselling sessions. The mothers in the intervention group attended six sessions of counselling with a health literacy approach in addition to counselling on routine pregnancy care. The control group attended counselling sessions on safe pregnancy care and received a training package containing all the subjects discussed in the intervention group. The Lifestyle Questionnaire and the Iranian Health Literacy Questionnaire were completed by the mothers at the beginning and at the end of the sessions as well as three weeks after the sessions. The data obtained were analyzed in SPSS-19.

**Results:** According to the study findings, the scores of lifestyle (P=0.8) and health literacy (P=0.423) showed no significant differences between the intervention and control groups before the intervention. Significant differences were, however, observed in the mean scores of lifestyle and health literacy between the two groups immediately and three weeks after the intervention. Comparing the means showed a higher increase in the mean scores in the intervention group (P<0.001).

**Conclusions:** Providing counselling services by midwives can significantly help modify mothers’ unhealthy lifestyle choices and increase their health literacy; therefore, reducing maternal and neonatal consequences, especially in high-risk pregnancies.

**Trial registration number:** IRCT2017021427728N3

**Trial registry:** Iranian Registry of Clinical Trials

**Trial registration date: **5th April 2017

## Introduction

Although pregnancy is a normal physiologic phenomenon, it can deviate from its normal path and cause disability, or even maternal and fetal mortality ^1^. Gestational diabetes is a major disorder that causes numerous complications in the mother and her baby. Gestational diabetes is a metabolic disorder that emerges or is diagnosed for the first time with carbohydrate intolerance during pregnancy and is considered the most common gestational complication ^2^. The prevalence of gestational diabetes differs in different regions, which can be explained by factors, such as ethnic diversity, differences in lifestyle and nutrition and different screening protocols ^3^. This disease influences maternal and fetal health and causes the emergence of numerous undesirable outcomes ^4^. These complications include hypertension, preeclampsia, urinary tract infections, hydramnios, surgical interventions, the risk of developing type 2 diabetes in mothers in the future, and macrosomia followed by an increased risk of birth trauma, congenital anomalies, childhood obesity and growth disorders in neonates ^5^.

Many factors are associated with gestational diabetes, including social determinants of health ^6^. Lifestyle is one of the main factors that can play a key role in preventing or treating gestational diabetes. Research suggests serious relationships between developing diabetes and lifestyle risk factors, such as smoking, inappropriate nutrition, drinking, obesity and limited physical activity ^7, 8^. Nowadays, more than 70% of diseases are believed to be somewhat related to people’s lifestyle and many illnesses appear to be directly or indirectly caused by lifestyle or at least be exacerbated or sustained by lifestyle ^9, 10^. Gestational diabetes prevention strategies have recently focused on promoting healthy lifestyles in patients by encouraging physical activity and promoting healthy nutrition ^11^. Nevertheless, modifying lifestyle requires adequate information in this regard ^12^. In fact, the decisions made by people and their performance regarding lifestyle behaviors depends on their level of literacy, which exerts a key effect on preventing and controlling chronic diseases such as diabetes ^13^. Today, health literacy improves health behaviors, creates a healthy lifestyle and promotes the quality of life ^14– 16^. Health literacy refers to one’s capacity for acquiring, interpreting and understanding primary health information and services required for proper health decision making ^17^. Enhancing the awareness and preparedness during pregnancy helps the mother pass this stage of life with fewer complications. As a result, pregnancy provides a good opportunity for teaching and counseling pregnant women and making them aware of the advantages of having a healthy lifestyle ^18^. Given the importance of pregnant women as a vulnerable social class, the fifth goal of United Nations Millennium Development Goals, i.e. a 75% reduction in maternal mortality through improving mothers’ health ^19^, and the significance of gestational diabetes as the most common pregnancy complication and a threat to the mother’s and baby’s health, the present study was performed to examine the effect of a health literacy approach to counselling on the lifestyle of women with high-risk pregnancy and gestational diabetes.

## Methods

The present randomized controlled clinical trial was conducted using a parallel design. The study population comprised pregnant women with gestational diabetes presenting to Alborz and Kamali hospitals in Karaj, Iran in April 2017.

The present research was approved by the Ethics Committee of Alborz University of Medical Sciences and Health Services on 4 March 2017 (Abzums.Rec.1395.146), and registered in the Iranian Registry of Clinical Trials ( IRCT2017021427728N3) on 5 ^th^ April 2017. A completed CONSORT checklist can be found in Supplementary File 1. 

## Subjects

**Inclusion criteria:** The eligible candidates comprised 18–35-year-old Iranian women with gestational diabetes, whose disease was first diagnosed using the FBS, OGTT or GCT tests, as per the criteria of the World Health Organization ^20^. The exclusion criteria consisted of being absent from, at most, two counseling sessions, having other physical or mental co-morbidities, such as cardiac, renal and thyroid disorders, and unwillingness to participate in the study.

After going to prenatal clinics of Alborz and Kamali Hospitals for routine check-ups, the researcher (MG) identified eligible mothers and explained the study objectives to them and asked them to sign an informed consent form if they were willing to participate. Convenience sampling was first used to select the subjects. They were then assigned to the intervention and control groups based on randomized blocks of four. Each “block” had a specified number of randomly ordered treatment assignments.

### Sample size calculation

Based on the formula for calculating the difference between two ratios and given a probability of proper lifestyle in 10% of the subjects in the intervention group (with gestational diabetes) and its improvement to 30%, the sample size was calculated as 84, i.e. n=42 in each group.

$$n2=\left\lceil z1-\alpha /2\right\rceil \sqrt{2p(1-p)}+z1-\beta \sqrt{p1(1-p1)+p2(1-p2)}]2/p1-p2$$

α=5 % β=20 % P1=10% P 2=3% n1/n2=1 n=42

### Intervention

The intervention group received counselling on routine pregnancy care and a health literacy approach to counselling for modifying lifestyle. The control group received counselling on routine pregnancy care as per the safe national maternal protocol of Ministry of Health and Medical Education of Iran as well as a training package containing all the subjects discussed in the intervention group. The sessions were taken by MG, MT. More information about the training sessions are included in Supplementary File 2.

Both groups attended six 1.5-hour sessions, once a week. To prevent cross talk by participants, the counselling sessions for the control and intervention groups were held on different days. The researcher followed up the mothers by phone.

The content of sessions in the intervention group was presented using a health literacy approach according to the protocol for the care of mothers with gestational diabetes approved by Ministry of Health and Medical Education of Iran as follows:

*Session one:* Introducing the members of the group to one another and the group leader, establishing a positive relationship, stating the group rules and creating motivations for the active participation and timely attendance in the sessions, explaining gestational diabetes and attitudes towards it and the role of a proper lifestyle in coping with gestational diabetes.

*Session two:* Counselling on self-awareness skills, understanding the status quo, explaining the suffering and difficulties they experienced, introducing and learning how to use insulin, injection-associated problems, attitudes towards insulin treatment and the ways of coping with these attitudes and illustrating a favorable status.

*Session three:* Counselling on health-related skills, including understanding basic information about nutrition, essential diets, understanding the effects of nutrition on improving the status, understanding basic information about physical exercise and its effects on improving maternal and fetal status, and explaining the capacity of performing exercises and the types of exercise allowed during pregnancy.

*Session four:* Counselling on the concepts associated with emotional skills, including understanding the fear and stress caused by the effect of the disease on the fetus and the body, the subject’s appearance and the disease survival after pregnancy and the ways of coping with it.

*Session five:* Understanding and counselling on the concepts associated with communication skills, understanding the effect of family and social supports, comprehending basic information about incorrect health behaviors and their effects on the mother and the fetus, understanding the effect of the support provided by the spouse, family and society on these behaviors and introducing services associated with pregnancy and screening.

*Session six:* Summarizing the presented subjects, practicing the counseled skills, responding to questions and receiving feedback on the classes held.

At the beginning, the end and three weeks after the sessions, Iranian Health Literacy Questionnaire (IHLQ) and Lifestyle Questionnaire (LSQ) were distributed among both groups and they were asked to complete them. If the patient was illiterate, the researcher completed the questionnaire by an interview.

### Data collection

The data collection tools comprised the IHLQ, the LSQ and a sociodemographic checklist ( Supplementary File 3).

The 53-item IHLQ contains 9 subscales, including access to health information access, health information use, reading skills, comprehension skills, assessment and judgment skills, decision-making and communication skills, health knowledge, individual empowerment and social empowerment. The validity and reliability of IHLQ were confirmed by Haghdoost *et al*. in 2014 ^21^.

The 70-item LSQ contains 10 dimensions of health, physical health, sports and fitness, weight management and nutrition, disease prevention, mental health, spiritual health, social health, avoidance of drugs, alcohol and opiates, accident prevention and environmental health. Lali *et al.* confirmed the validity and reliability of this questionnaire in Isfahan, Iran, in 2008–2009 academic year ^10^.

### Data analysis

The data collected were analyzed in SPSS-19 using independent t-test, Mann-Whitney test, Fisher’s exact test and Chi-square test.

## Results

**Figure 1.**
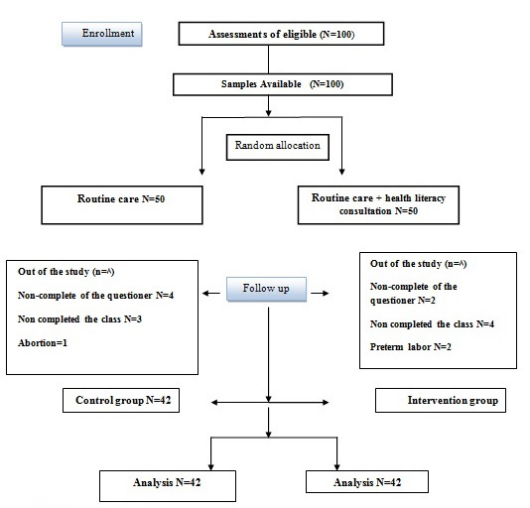
CONSORT flow diagram.

A total of 100 subjects were initially recruited, and 16 were excluded during the study: 8 subjects were excluded in the intervention group, including 2 for failing to complete the questionnaires, 4 for being absent for more than two sessions, and 2 for preterm delivery at a 25-week and 29-week gestational age; 8 subjects were also excluded from the control group, including 4 for failing to complete the questionnaires, 3 for failing to attend the sessions and 1 for abortion at 16 weeks of pregnancy. Therefore, the study ended with 84 subjects ( Figure 1).

The present study investigated 84 mothers with gestational diabetes in two groups, who were examined until the end of the study. After examining the normality of the study variables, no significant differences were observed between the two groups in terms of mean age, level of education, body mass index (BMI), occupational status and gestational age, and the two groups were therefore matched in terms of the cited variables ( Table 1).

**Table 1.** Distribution of demographic characteristics in women with gestational diabetes presenting to selected health centers in Alborz province in 2017.

Demographic factors		Control group	Intervention group	P value
Mean ±SD				
Maternal age		30.78±3.82	31.5±4.36	0.734
Maternal weight before pregnancy		70.21±10.88	71.02±8.97	0.71
Gain weight in pregnancy		6.45±2.34	6.80±3.31	0.34
BMI		27.50±3.554	28.11±3.267	0.68
Gestational age by sonography		23.62±6.23	22.81±8.38	0.93
N (%)				
Maternal job	House keeper	39 (92.9)	38 (90.5)	0.5
	Employment	3 (7.1)	4 (9.5)	
Maternal education	Diploma	36 (85.7)	34 (81.0)	0.53
	BS	6 (14.3)	7 (16.7)	
	MS	0 (0)	1 (2.4)	

According to the study findings, no significant differences were observed between the two groups in terms of the mean score of lifestyle before the intervention. Therefore, the two groups were matched before the intervention in terms of lifestyle. The two groups exhibited significant differences in the mean score of lifestyle after the intervention. This mean score showed a significant increase immediately and three weeks after the intervention, and this increase was higher in the intervention group ( Table 2).

**Table 2.** Comparing the overall mean score of lifestyle in the control group and the intervention group before, immediately after and three weeks after (follow-up) the intervention in women with high-risk pregnancy presenting to selected health centers in Alborz province in 2017.

		Before intervention	Immediately After intervention	3 weeks after intervention	P value *
Physical health	Intervention group	14.12±2.81	19.28±2.17	20.83±1.58	P<0.001 F=173.96
	Control group	13.95±2.80	16.05±3.02	16.57±3.11	
Sports and fitness	Intervention group	10.07±3.39	15.93±1.93	16.90±1.95	P<0.001 F=208.14
	Control group	8.74±3.15	10.40±2.81	10.95±3.099	
Weight management and nutrition	Intervention group	12.28±2.40	17.40±1.69	18.19±1.435	P<0.001 F=130.70
	Control group	12.76±3.39	13.88±2.68	13.71±2.75	
Disease prevention	Intervention group	15.74±2.58	18.00±1.89	18.81±1.52	P<0.001 F=43.82
	Control group	15.64±2.32	16.21±2.65	16.28±2.31	
Mental health	Intervention group	14.238±3.69	17.50±2.37	18.17±1.94	P<0.001 F=43.82
	Control group	13.83±3.77	14.81±3.44	14.93±3.42	
Spiritual health	Intervention group	14.36±6.31	14.93±2.38	15.48±1.89	P<0.001 F=1.46
	Control group	13.12±3.28	13.26±2.46	13.40±2.18	
Social health	Intervention group	15.57±3.26	18.67±1.54	18.93±1.58	P<0.001 F=53.28
	Control group	15.57±3.52	15.86±2.87	16.55±2.57	
Avoidance of drugs, alcohol and opiates	Intervention group	14.71±3.34	17.00±0.91	17.33±0.95	P<0.001 F=12.54
	Control group	15.14±3.59	15.45±3.39	15.71±2.81	
Accident prevention	Intervention group	18.57±3.46	20.90±2.16	21.78±1.64	P<0.001 F=24.02
	Control group	19.33±2.99	19.86±3.17	19.81±2.91	
Environmental health	Intervention group	17.78±3.48	18.62±3.12	20.98±10.10	P=0.169 F=1.91
	Control group	18.90±3.11	18.26±2.88	18.00±2.84	
**Life style**	Intervention group	144.67±21.53	175.64±12.84	184.00±12.24	P<0.001 F=208.87
	Control group	143.52±19.92	151.33±18.33	153.40±16.56	

*P-values are comparing the difference in scores of lifestyle in three phases, before, immediately after and 3 weeks after intervention, between the intervention and the control groups.

No significant differences were observed between the two groups in terms of the mean score of health literacy before the intervention, and the two groups were therefore matched in this regard. The two groups exhibited significant differences in the mean score of health literacy immediately and three weeks after the intervention, and this increase was significant in the intervention group ( Table 3). 

**Table 3.** Comparing the overall mean score of health literacy in the control group and the intervention group before, immediately after and three weeks after (follow-up) the intervention in women with high-risk pregnancy presenting to selected health centers in Alborz province in 2017.

Health literacy	Before intervention	After intervention	3 weeks after intervention	
Intervention group	9.95±2.52	14.44±1.29	13.17±1.77	F=278.69 P<0.001 *
Control group	10.36±2.14	11.66±1.89	11.34±1.98	

*P-value is comparing the difference in scores of health literacy in three phases, before, immediately after and 3 weeks after intervention, between the intervention and the control groups.

Sociodemographic characteristics, and results from the IHLQ and LSQ questionnaires, before, immediately after and 3 weeks after intervention for control and intervention groups Click here for additional data file. Copyright: © 2018 Gharachourlo M et al. 2018 Data associated with the article are available under the terms of the Creative Commons Zero "No rights reserved" data waiver (CC0 1.0 Public domain dedication).

## Discussion

Gestational diabetes is a common disorder during pregnancy, which can negatively affect prenatal outcomes and is associated with lifestyle risk factors. Modern prevention methods of gestational diabetes emphasize modifying lifestyle risk factors ^18, 22^.

The present study findings suggest no significant differences between the two groups before counselling in terms of the score of lifestyle. In other words, the two groups presented the same level of lifestyle upon entering the study; however, immediately after and three weeks after the intervention, a higher increase was observed in the mean scores of variables, such as physical health, sports and fitness, weight management and nutrition, disease prevention, mental health, avoidance of drugs, alcohol and opiates, accident prevention and the overall lifestyle of the mothers, in the intervention group compared to those in the control group, although no significant differences were observed between the two groups in spiritual health and environmental health.

In 2011, Luoto *et al*. found that interventions such as counselling can improve lifestyle quality in pregnant patients at risk of developing gestational diabetes ^23^. Numerous studies have explained the effect of interventions on modifying lifestyle, and their results are consistent with those obtained in the present study. In 2015, Khadivzadeh *et al*. found that educational lifestyle interventions can help promote self-care in patients with gestational diabetes ^24^. Babaei *et al*. (2016) ^25^ and Korpi-Hyövälti *et al.* (2012) reported that individual counseling conducted by experts as part of lifestyle interventions modifies nutrition and sports dimensions of lifestyle ^26, 27^. A study conducted by Babei *et al.* titled “the effect of educational intervention of lifestyle modification on blood pressure control in patients with hypertension” showed that educational programs can modify and increase the score of lifestyle dimensions, including sports and fitness, weight management and nutrition and mental health, although these interventions were found not to affect spiritual health ^25^.

Before the intervention, no significant differences were observed between the two groups in the score of health literacy, and the two groups were matched in terms of the level of health literacy. The mean score of health literacy, however, showed a higher increase immediately and three weeks after the intervention in the intervention group compared to that in the controls. Given that the two groups were matched in terms of demographic characteristics and level of health literacy, the findings suggest the effect and role of counseling and the counselor midwife in improving the level of health literacy in mothers with gestational diabetes. This finding is consistent with the results of the studies conducted by Tol *et al*. ^28^ and Kandula *et al.* ^29^. These researchers found that education and counselling increase the score of health literacy in diabetics with any levels of health literacy. Luoto *et al*. reported that interventions at the level of patient communication skills can improve the awareness, literacy and clinical indicators of diabetics ^23^. Today, many health problems and human social needs can be solved using advisors as facilitators for knowledge based on useful relationships. ^30^.

The strength of the present study was the fact that the intervention and control groups matched in terms of some confounding demographic variables affecting the results, including age, level of education, BMI and gestational age, and the results obtained were not therefore affected by these variables. The researchers made efforts to use random allocation and match the subjects in both groups; however, the subjects’ lifestyle was impossible to be completely controlled and this was a limitation of the present research.

## Conclusion

The findings obtained from the present research showed that both routine pregnancy counselling and a health literacy approach to counselling causes an increase in the overall score of lifestyle and health literacy. As members of the health system and the main supervisors of pregnant women, midwives have the closest relationship with these women and are in charge of providing them with care services and necessary recommendations during pregnancy. They can therefore take more effective steps towards improving mothers’ health and pregnancy outcomes by applying correct counselling principles for resolving mothers’ problems.

## Ethical statement

The present research was approved by the Ethics Committee of Alborz University of Medical Sciences and Health Services on 4 March 2017 (Abzums.Rec.1395.146). Written informed consent was obtained from all subjects for participation in the study.

## Data availability

The data referenced by this article are under copyright with the following copyright statement: Copyright: © 2018 Gharachourlo M et al.

Data associated with the article are available under the terms of the Creative Commons Zero "No rights reserved" data waiver (CC0 1.0 Public domain dedication). 

Dataset 1: Sociodemographic characteristics, and results from the IHLQ and LSQ questionnaires, before, immediately after and 3 weeks after intervention for control and intervention groups. DOI, 10.5256/f1000research.13838.d195054 ^31^

## Supplementary material

Supplementary File 1: CONSORT checklist.

Supplementary File 2: Information about the control and intervention group sessions.

Supplementary File 3: Sociodemographic checklist.
