# Supplementary material for: The effect of a health literacy approach to counselling on the lifestyle of women with gestational diabetes: A clinical trial
Source: F1000Res. 2018 Mar 6;7:282. [Version 1] doi: 10.12688/f1000research.13838.1 (PMC5854987; doi:10.12688/f1000research.13838.1)
Supplement: Supplementary file 3 [file f1000research-7-15041-s0002.tgz › 6ec74121-653f-48a2-bc56-2d0f0a71f7ab.docx]

**Control group**

Session 1 Introduction to pregnancy and its symptoms and personal and psychological health

Session 2 Personal and psychological health

Session 3 Sexual health

Session 4 Nutrition and nutritional supplements

Session 5 Introduction to risk factors

Session 6 Problem-solving training Breastfeeding training and postpartum care instructional booklets

**Intervention group**

Sessions one: explaining gestational diabetes and attitudes towards it and the role of a proper lifestyle in coping with gestational diabetes.

Session two: counseling on self-awareness skills, understanding the status quo, explaining the suffering and difficulties they experienced, introducing and learning how to use insulin, injection-associated problems, attitudes towards insulin treatment and the ways of coping with these attitudes and illustrating the favorable status.

Session three: counseling on health-related skills, including understanding basic information about nutrition, essential diets, understanding the effects of nutrition on improving the status, understanding basic information about physical exercise and its effects on improving maternal and fetal status and explaining the capacity of performing exercises and the types of exercise allowed during pregnancy.

Session four: counseling on the concepts associated with emotional skills, including understanding the fear and stress caused by the effect of the disease on the fetus and the body, the subject’s appearance and the disease survival after pregnancy and the ways of coping with it.

Session five: understanding and counseling on the concepts associated with communication skills, understanding the effect of family and social supports, comprehending basic information about incorrect health behaviors and their effects on the mother and the fetus, understanding the effect of the support provided by the spouse, family and society on these behaviors and introducing services associated with pregnancy and screening.

Session six: summarizing the presented subjects, practicing the counseled skills, responding to questions and receiving feedback on the classes held
