# Supplementary material for: The effect of a health literacy approach to counselling on the lifestyle of women with gestational diabetes: A clinical trial
Source: F1000Res. 2018 Mar 6;7:282. [Version 1] doi: 10.12688/f1000research.13838.1 (PMC5854987; doi:10.12688/f1000research.13838.1)
Supplement: Supplementary file 4 [file f1000research-7-15041-s0003.tgz › 51cdaf0c-0342-4e42-b2df-197f957ae64e.pdf]

**Socio Demographic**

Dear participants

I am a graduate student in midwifery counseling of Alborz University of Medical sciences, I want to do research on "The effect of counseling on the health literacy approach on women's lifestyles with high risk pregnancies" in Alborz province. Sincerely, Thank you for your cooperation, and the information you receive will be kept secret.

. Please read the following and answer them according to your living conditions.

| | |
|---|---|
| Husbands age ........................................<br>Ethnic Husbands ............................................... | Age .........................<br>Ethnic ............................ |
| Mothers job 1=employed 2=housekeeper<br>Husbands job 1=clerk 2=free job 3=worker 4= unemployed | Education<br>Husbands education |
| Last menstrual period | Gestational age |
| Number of delivery<br>Live child number | Number of pregnancy |
| Contraception type | Number of Intra uterine fetal death<br>Number of abortion |
| Age of marriage | 1=less than 18 2=18-28 3=28-35 4=more than 35 |
| High<br>BMI | Weight before pregnancy |
| History of gestational diabetes 1= no 2= yes | Increase weight |
| History of in family<br>1= no 2= yes | History of disease 1=no 2=yes |
| History of preterm birth 1=no 2=yes | History of low birth weight 1=no 2=yes |
| History of intra uterine growth retardation 1=no 2=yes | History of abortion 1=no 2=yes |
| History of fetal macrozomia 1=no 2=yes | First FBS in pregnancy 1=92-125 2=more than 125 |
| Kind of treatment 1=diet 2=oral drug 3=insulin | Multivitamin intake in pregnancy 1=no 2=yes |
