# Supplementary material for: The effect of a health literacy approach to counselling on the lifestyle of women with gestational diabetes: A clinical trial
Source: F1000Res. 2018 Mar 6;7:282. [Version 1] doi: 10.12688/f1000research.13838.1 (PMC5854987; doi:10.12688/f1000research.13838.1)
Supplement: Sociodemographic characteristics, and results from the IHLQ and LSQ questionnaires, before, immediately after and 3 weeks after intervention for control and intervention groups [file f1000research-7-15041-s0000.tgz › cba42d26-a545-40d5-8359-05045c8f8119_Dataset_1.zip!/Dataset 1/Coding scheme.docx]

| Group | 1=control 2= intervention |
| --- | --- |
| Hage | Husbands age |
| Hethnic | Husbands ethnic |
| Heducation | Husbands education 1=less than diploma 2=diploma-MS 3=MS-PHD |
| Job | Mothers job 1=employed 2=housekeeper |
| Hjob | Husbands job 1=clerk 2=free job 3=worker 4= unemployed |
| Gage | Gestational age |
| gravid | Number of pregnancy |
| para | Number of delivery |
| LC | Number of living childs |
| chidnumber | Number of child |
| abortion | Number of Abortion |
| IUFD | Intra uterine fetal death |
| infertility | History of infertility 0= no 1=yes |
| interval | Interval of pregnancy |
| contraceptive | Type of contraceptive that was used |
| Agemarriage | Age of marriage 1=less than 18 2=18-28 3=28-35 4=more than 35 |
| pweight | Weight before pregnancy |
| High | High of Mother |
| increasweight | Increase weight during pregnancy |
| Hdisease | History of disease 1=no 2=yes |
| Hpretermbirth | History of preterm birth 1=no 2=yes |
| Hlbw | History of low birth weight 1=no 2=yes |
| HIUGR | History of intra uterine growth retardation 1=no 2=yes |
| Hmacrozomia | History of fetal macrozomia 1=no 2=yes |
| Hgestationaldiabetes | History of gestational diabetes 1= no 2= yes |
| Habortion | History of abortion 1=no 2=yes |
| HFdiabetes | Family history of diabetes 1=no 2=yes |
| drug | Multivitamin intake in pregnancy 1=no 2=yes |
| Controldiabetes | Kind of treatment 1=diet 2=oral drug 3=insulin |
| fFBS | First FBS in pregnancy 1=92-125 2=more than 125 |
| LSQ A | Lifestyle questioner before intervention question 1 up to question 70 |
| LSQ B | Lifestyle questioner after intervention question 1 up to question 70 |
| LSQ C | Lifestyle questioner 3weeks after intervention question 1 up to question 70 |
| IHLQA | Iranian health literacy questioner before intervention question 1 up to question 62 |
| IHLQB | Iranian health literacy questioner after intervention question 1 up to question 62 |
| IHLQC | Iranian health literacy questioner after 3weeks of intervention question 1 up to question 62 |
