# Supplementary material for: The effect of a health literacy approach to counselling on the lifestyle of women with gestational diabetes: A clinical trial
Source: F1000Res. 2018 Mar 6;7:282. [Version 1] doi: 10.12688/f1000research.13838.1 (PMC5854987; doi:10.12688/f1000research.13838.1)
Supplement: Peer review history

# Peer review: Referee response for version 1

**DOI:** 10.5256/f1000research.15041.r31591

**Published:** 2018-04-03

**Authors:** Fariba Ebtekar

**Affiliations:** 1 Department of Midwifery, Faculty of Nursing and Midwifery, Kurdistan University of Medical Sciences, Sanandaj, Iran

1. The problem statements agreed with the title and seemed to be significance. Several appropriate references were used in the introduction section, but the literature was not clearly visible to the reader, and it required several conflicting findings about the effect of Health literacy on life style.
2. The aim of this study is clearly stated, however, it is better to remove "high risk pregnancy "in the last sentences of the introduction section.
3. In methodology section, the instruments were explained, and their reliability and validity were stated. The population used was adequate. The counselling sessions were explained clearly.
  It is better to clarify the number of participants in intervention and control groups from each hospital. The statistical techniques were stated but no discussion was given in this particular section.
4. In the results section, the findings were presented by appropriate figure and tables according to aim of the study.
5. In the discussion section, the findings of other studies supported the results of the present study but the conflicting findings were not stated.
6. The conclusion section was stated clearly that included summary of the main findings and recommendation but the recommendation for further researches was not given.
7. In the Socio Demographic check list it was better “weight gain” written instead of increase weight.

I have read this submission. I believe that I have an appropriate level of expertise to confirm that it is of an acceptable scientific standard.

# Peer review: Referee response for version 1

**DOI:** 10.5256/f1000research.15041.r31590

**Published:** 2018-03-15

**Authors:** Mojgan Javadnoori

**Affiliations:** 1 Reproductive Health Promotion Research Centre, School of Nursing and Midwifery, Ahvaz Jundishapur University of Medical Sciences, Ahvaz, Iran

Dear authors,

 Your manuscript addresses an important aspect of caring for high risk pregnancies, however some suggestions may be considered:

  

1. Introduction should clarify the gap in literature about interventions on lifestyle and their effects on pregnancy outcome. In the other words, what is gap in knowledge about this topic that your study aimed to answer it?
2. Please give explanations about this counselling approach (health literacy approach). Is your intervention a counselling or an education?
3. What does the “parallel design” mean? Please explain it.
4. The value of P for comparing before and after of each group is not mentioned in the tables.
5. It is important that, " no significant differences were observed between the two groups in terms of demographic variables", or life style, or health literacy, “ Therefore, the two groups were matched before the intervention in terms of lifestyle” ……  IS NOT “matching process”. Matching process is used in case-control design. “Random allocation” is utilized to control such confounding variables in randomized control trials. So it should be correct throughout the results and discussion; sentences such as:  “The strength of the present study was the fact that the intervention and control groups matched in terms of …”
6. The first sentence in conclusion part is not highlighted in results because the p value In tables, is for comparing two groups no before and after in one group.
7. The first paragraph of discussion can be deleted.
8. The title of tables is very long and contains unnecessary explanations.
9. It is suggested to change the word “subjects” to “participants”.
10. The last sentence of the background in abstract should be remove to methods.
11. The content of counselling approach for intervention group, vs routine care and package for control group is not clear. “The control group received counselling on routine pregnancy care as per the safe national maternal protocol of Ministry of Health and Medical Education of Iran”. Is this protocol the same that was used as counselling for routine pregnancy care? Please describe it more clearly.
12. What is the novelty of your study findings?

I have read this submission. I believe that I have an appropriate level of expertise to confirm that it is of an acceptable scientific standard.

# Peer review: Referee response for version 1

**DOI:** 10.5256/f1000research.15041.r31588

**Published:** 2018-03-14

**Authors:** Shirin Djalalinia

**Affiliations:** 1 Noncommunicable Diseases Research Center, Endocrinology and Metabolism Population Sciences Institute, Tehran University of Medical Sciences, Tehran, Iran

Gharachourlo et al performed an interesting clinical trial about the effect of a health literacy approach to counselling on the lifestyle of women with gestational diabetes, enrolled 42 eligible 18–35-yearold Iranian women with gestational diabetes in each groups. They concluded that providing counselling services by midwives can significantly promote the lifestyle patterns and increase their health literacy, especially in high-risk pregnancies.

 The field of gestational diabetes consider as one of the most important priorities of maternal health and designing and development of the manuscript are interesting.

 I think the paper may therefore be suitable for publication after some minor changes that I suggested below:

- Please rename 'subjects' to 'participant'.
- In the Introduction section; please provide the gap of related evidence and briefly point to justification of conduction of present study in these regards.
- In the Methods section; refer to your approaches in data analyzing even used soft wares.
- The strengths aspects of investigation could be discussed in more details.
- Further subjects could be providing for more complementary researches in related domains.

I have read this submission. I believe that I have an appropriate level of expertise to confirm that it is of an acceptable scientific standard.
